# Rare primary malignant melanoma of the esophagus with gastric cardia adenocarcinoma: A case report

**DOI:** 10.1097/MD.0000000000044988

**Published:** 2025-10-10

**Authors:** Yu Zhou, Li Li Jin, Wei Wei Min, Qi Bin Shen

**Affiliations:** a Huzhou Central Hospital, The Affiliated Central Hospital of Huzhou University, Huzhou, Zhejiang Province, China; b Department of Central Laboratory, Huzhou Central Hospital, Affiliated Central Hospital of Huzhou University, Huzhou, Zhejiang Province, China; c Department of Cardiothoracic Surgery, Huzhou Central Hospital, Affiliated Central Hospital of Huzhou University, Huzhou, Zhejiang Province, China.

**Keywords:** gastric adenocarcinoma, primary malignant melanoma of the esophagus, radiotherapy

## Abstract

**Rationale:**

This case report presents an unusually rare instance of primary malignant melanoma of the esophagus (PMME) occurring concurrently with gastric cardia adenocarcinoma. It emphasizes diagnostic pitfalls in amelanotic melanoma and provides evidence supporting the role of adjuvant radiotherapy in achieving prolonged disease-free survival for this aggressive malignancy.

**Patient concerns:**

A 60-year-old male with chronic tobacco and alcohol use presented with a 2-week history of progressive dysphagia. Endoscopy identified an obstructive esophageal mass and a gastric cardia ulcer, initially misdiagnosed as poorly differentiated carcinomas due to the absence of melanin pigmentation.

**Diagnoses:**

The patient received a diagnosis of PMME in conjunction with gastric cardia adenocarcinoma.

**Interventions:**

Transthoracic esophagectomy with lymphadenectomy followed by adjuvant radiotherapy (5000 cGy/DT/25F) targeting the surgical bed and regional lymphatics.

**Outcomes:**

No recurrence or metastasis was observed over 7 years posttreatment, surpassing typical survival outcomes for PMME.

**Lessons:**

Amelanotic PMME poses significant diagnostic challenges due to its resemblance to poorly differentiated carcinoma, necessitating immunohistochemical confirmation (S-100, HMB-45, Melan-A). This case highlights the potential survival benefit of combining surgery with adjuvant radiotherapy in PMME, contrasting with historical outcomes in cases managed by surgery alone. Clinicians should maintain a high index of suspicion for synchronous malignancies in high-risk patients, particularly those with chronic carcinogen exposure. Emerging therapies, including immune checkpoint inhibitors targeting programmed cell death-1/CTLA-4 or NF-1 mutation-driven pathways, warrant exploration in PMME management. The unique lymph node metastasis pattern underscores the importance of comprehensive nodal sampling and tailored adjuvant strategies in dual-primary malignancies.

## 1. Introduction

Malignant melanoma primarily manifests in the skin, with primary malignant melanoma of the esophagus (PMME) being a rare occurrence. PMME is more prevalent than melanoma metastasis to the esophagus.^[1]^ The melanocytes present in the esophagus are believed to originated from either the abnormal migration of melanoblasts from the neural crest or from APUD cells of the intestinal neuroendocrine system.^[2]^ Surgical intervention is the most common form of treatment for affected patients, however, the prognosis remains grim, with a 5-year survival rate ranging from 4% to 37%.^[3]^ Esophagogastric junction cancer is classified into 3 types, with Siewert type III often being associated with gastric cardia cancer. Among them, incidence rates of adenocarcinoma are rising rapidly, due to the decrease levels of *Helicobacter pylori* infection and increase in gastroesophageal reflux disease.^[4]^ Previous literature indicates that the occurrence of dual primary cancers is exceedingly rare and associated with a poor prognosis. This study aims to raise awareness of this condition by disseminating pertinent clinical experiences.

## 2. Case presentation

**Figure 1.**
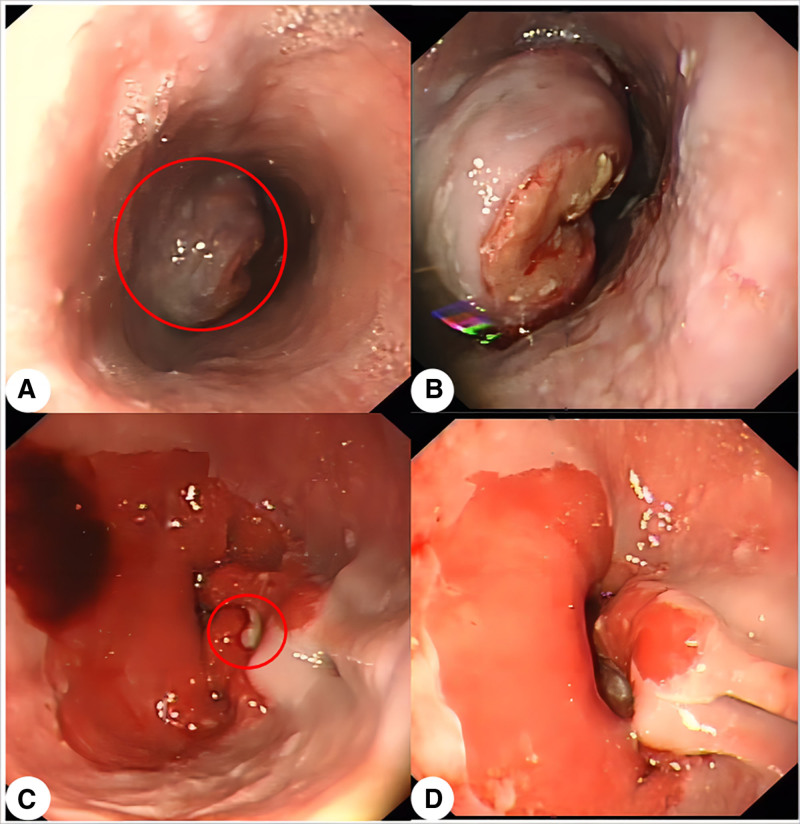
Endoscopic findings imaging (May 19, 2017). (A, B) A mass in the esophagus located 32 to 38 cm from the incisors, extends from the lumen and is usually identified as amelanotic melanoma (circle). (C, D) A 1.2-cm ulcer of the cardia (circle).

A 60-year-old male patient presented with a 2-week history of dysphagia following ingestion of solid food. The patient denied experiencing symptoms such as vomiting or melena. He had a long-standing history of tobacco (35 cigarettes/d) and Chinese liquor (312 mL/d) use. Endoscopic examination revealed the presence of a mass in the esophagus located 32 to 38 cm from the incisors, obstructing the lumen and impeding passage of the endoscope (Fig. 1A, B). Additionally, a 1.2-cm ulcer was visualized on the posterior lateral wall of the cardia, protruding into the stomach cavity (Fig. 1C, D). Pathological examination of the 2 lesions revealed that the esophageal mass was characterized by poorly differentiated carcinoma, while the gastric cardia ulcer exhibited features of moderately to poorly differentiated adenocarcinoma. No evidence of metastatic foci was identified in any other part of the body. Subsequently, a transthoracic esophagectomy approach was carried out to address both esophageal and gastric cardia cancers.

**Figure 2.**
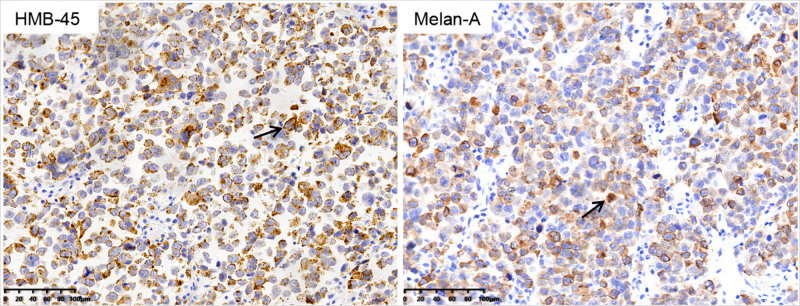
The images show immunohistochemical analysis of esophageal lesions, demonstrating positive staining for HMB-45 and Melan-A (×40; arrow).

**Figure 3.**
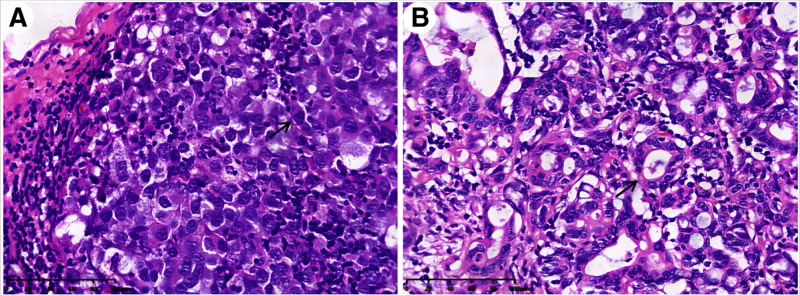
(A) The perigastric lymph node exhibit evidence of metastatic spread of malignant melanoma (H&E stain, ×40; arrow). (B) Another perigastric lymph node exhibits signs of metastatic adenocarcinoma dissemination (H&E stain, ×40; arrow).

**Figure 4.**
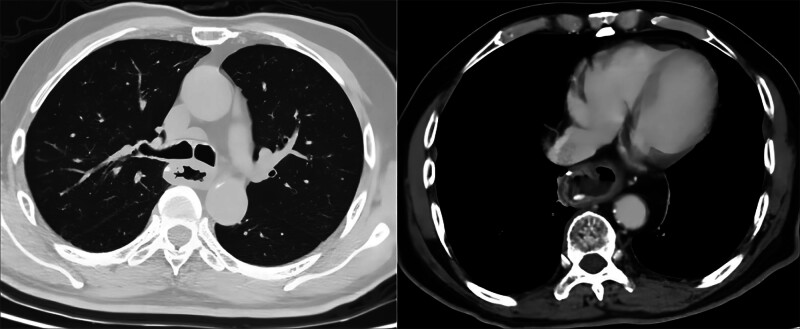
Images obtained during the postoperative reexamination conducted in May 2024.

The endoscopic evaluation revealed no alterations in pigmentation within the esophageal mass, which resulted in the exclusion of melanoma as a potential diagnosis. The postoperative pathology revealed that the esophageal mass had a maximum diameter of 6 cm and had infiltrated into the adventitial. Microscopic examination showed small, round cancer cells lacking melanin granules. Immunohistochemical (IHC) analysis demonstrated positive expression of HMB-45 and Melan-A (Figs. 2, S1–S4, Supplemental Digital Content, https://links.lww.com/MD/Q256). The gastric cardia ulcer was identified as a moderately to poorly differentiated adenocarcinoma measuring 1.7 cm × 1.5 cm, infiltrating the superficial muscular layer and involving the esophagus. Surgical lymph node dissection revealed the presence of 1 perigastric lymph node containing metastatic malignant melanoma (Fig. 3A), as well as another lymph node exhibiting metastasis from adenocarcinoma (Fig. 3B). Additional, malignant melanoma metastasis was present in the peritumoral and subcarinal lymph nodes. Metastasis was ruled out in other areas of the body, leading to a diagnosis of PMME combined with gastric cardia adenocarcinoma. Post-surgery, the patient received radiotherapy at a dose of 5000 cGy/DT/25F. As of the present time, there is no recurrence or metastasis (Fig. 4).

## 3. Discussion

Malignant melanoma presents as various subtypes, including skin cutaneous melanoma (SKCM), acral melanoma, mucosal melanoma (MM) and uveal melanoma. MM accounts for about 0.8% to 3.7%, is commonly localized in the nasopharynx, respiratory tract, gastrointestinal tract, and reproductive tract.^[5]^ PMME is rare and hundreds of cases have been reported in the literature. Furthermore, the co-occurrence of PMME combined with gastric cardia adenocarcinoma is even more uncommon. PMME predominantly manifests in the lower third of the esophagus, typically affecting individuals at 60.5 years, with a male to female ratio of 2:1.^[6,7]^ So, polypoid masses in the middle and lower third of the esophagus may be special types of cancer, specifically PMME. Several researchers have posited that hyperplastic epithelium or chronic esophagitis could potentially play a role in the development of melanocytosis in the basal layer of the epithelium.^[8]^ PMME generally does not result in obstruction, and a barium swallow examination typically identifies a polypoid or lobulated mass that extends into the lumen.^[9,10]^ This mass is characterized by a soft and fragile texture, which contributes to the manifestation of mild and delayed symptoms. Endoscopic examination revealed the presence of ulcers on the surface of the mass, along with adjacent satellite lesions (indicative of intramural metastasis) or melanotic macules.^[11]^

The pathological characteristics of PMME include basal melanocytes covered by squamous epithelium, focal adjacent junctional melanocytic activity or melanoma in situ,^[12]^ and a higher abundance of stromal blood vessels compared to squamous cell carcinoma.^[13]^ Diagnosis of PMME requires the exclusion of metastasis from other primary sites. Positive staining for S-100, HMB-45, melano-A and SOX-10, with HMB-45 being particularly specific due to its indication of active melanosome formation, is essential for confirming the diagnosis.^[14]^

In recent years, a significant number of studies have focused on the genomic analysis of PMME with the aim of discovering improvements in targeted therapies. Nevertheless, due to the rarity of the disease, there is currently no standardized treatment protocol in place. The BRAF gene mutation is frequently observed in SKCM, whereas NF-1, NARS, KIT, and SF3B1 gene mutations are more prevalent in PMME.^[15,16]^ NF-1 functions as a tumor suppressor gene, and mutations in NF-1 lead to abnormal activation of the MAPK pathway. Therefore, the use of cobimetinib may be beneficial for PMME patients with NF-1 mutations. A genomic analysis has indicated that individuals diagnosed with PMME might benefit from the current treatment used for common cutaneous melanoma.^[17]^ The first-line systemic therapy for melanoma is immunotherapy such as nivolumab, ipilimumab, and pembrolizumab according to the NCCN guidelines, with anti-programmed cell death-1 (PD-1) treatment showing potential efficacy in 20% to 30% of cases of mucosal malignant melanoma.^[18,19]^ Several case reports have documented the use of immunotherapy in achieving long-term survival in patients with PMME. And the utilization of anti-CTLA-4 antibody presents a novel therapeutic approach for malignant melanoma. Furthermore, several studies have indicated that the combination of PD-1 inhibitors with vascular endothelial growth factor inhibitors or ipilimumab demonstrates superior efficacy compared to monotherapy with PD-1 blockade.^[20,21]^

The impact of radiotherapy on PMME remains unverified; however, a review of the literature reveals instances where patients have experienced prolonged survival following a combination of surgical intervention and radiotherapy.^[22]^ Furthermore, the application of radiotherapy in the management of MM has demonstrated efficacy in achieving favorable local control.^[23]^ In the case presented, the patient underwent radiotherapy targeting both the surgical site and the lymphatic drainage region 2 months postoperatively, with no evidence of recurrence observed to date. This suggests that the combination of surgical treatment and radiotherapy may be advantageous for PMME. The rarity of PMME combined with adenocarcinoma is underscored by our retrieval of 3 cases through the literature,^[24–26]^ all of which underwent surgical treatment without the administration of adjuvant therapy and exhibited a poor prognosis (Table 1). This highlights the imperative need for postoperative adjuvant therapy in patients with this condition, irrespective of lymph node involvement.

**Table 1:** Information regarding cases obtained from the literature.

	Li J^[24]^	Zhou YB^[25]^	Pruyt M^[26]^
Gender	M	M	M
Age	64	71	58
Tumor site	E and G	E and EGJ	E
Therapy	Surgery	Surgery	Surgery
Overall survival	3 mo	–	8 mo
Lymph metastases	N	N	N

E = esophagus, G = gastric, EGJ = esophagogastric junction.

PMME, particularly amelanotic malignant melanomas, is a rare and highly aggressive tumor that can be easily misdiagnosed as poorly differentiated carcinoma. Therefore, immunohistochemical analysis is crucial for the diagnosis of poorly differentiated esophageal cancer. Additionally, there have been reported cases of PMME coexisting with other primary tumors, although the underlying mechanism of this occurrence remains unclear.

## 4. Conclusion

This study presents a case of primary malignant melanoma of the esophagus (PMME) concomitant with gastric cardia adenocarcinoma, featuring a unique lymph node metastasis pattern, 1 perigastric lymph node exhibited metastasis of malignant melanoma, while another showed adenocarcinoma metastasis. The patient underwent surgical intervention followed by adjuvant radiotherapy, resulting in a disease-free survival of over 7 years. The aggressive nature of PMME is associated with a generally unfavorable prognosis. This case provides valuable insights for management practices and enhances the understanding of dual primary malignant tumors.

## Acknowledgments

We express our sincere gratitude for the invaluable technical support provided by the Pathology Department at HuZhou Central Hospital.

## Author contributions

**Conceptualization:** Wei Wei Min, Qi Bin Shen.

**Data curation:** Wei Wei Min.

**Writing** – **original draft:** Yu Zhou.

**Writing** – **review & editing:** Yu Zhou, Li Li Jin.

## Supplementary Material


