# Supplementary material for: Rare primary malignant melanoma of the esophagus with gastric cardia adenocarcinoma: A case report
Source: Medicine (Baltimore). 2025 Oct 10;104(41):e44988. doi: 10.1097/MD.0000000000044988 (PMC12517885; doi:10.1097/MD.0000000000044988)

Fig S1: Immunohistochemical staining with HMB-45 antibody (x5). The specimen displayed on the left side of the image represents a surgically excised esophageal mass, whereas the tissue on the right corresponds to melanoma tumor tissue.

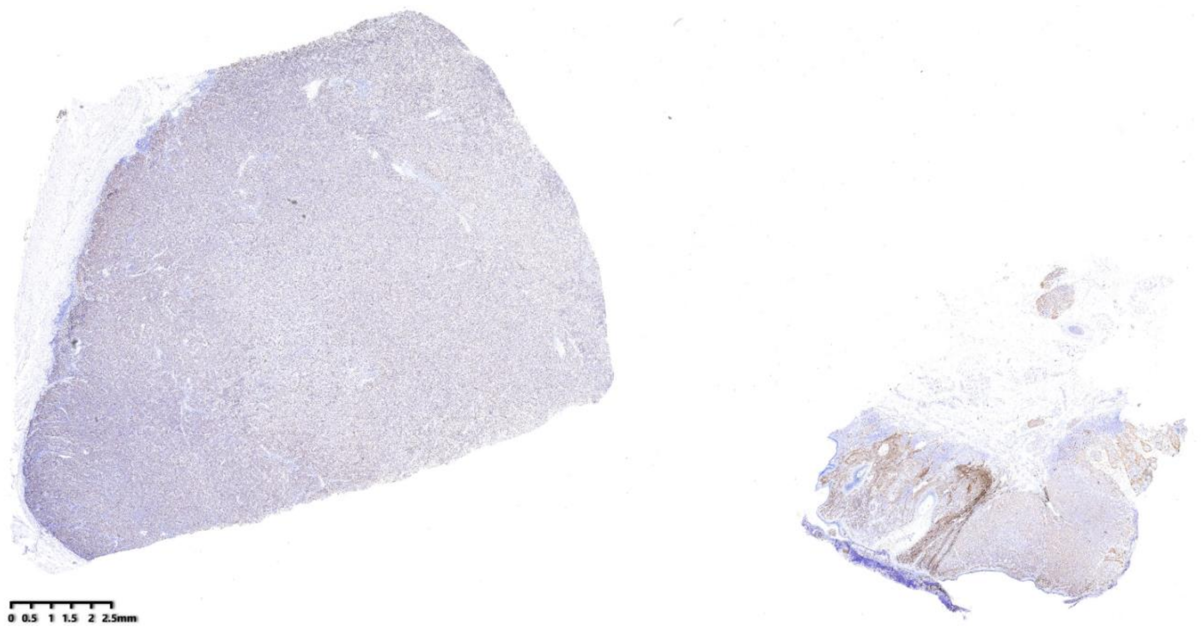

Fig S2: Immunohistochemical staining with HMB-45 antibody (x40). (a) The esophageal mass (arrow). (b) The positive control of melanoma tumor tissue (arrow).

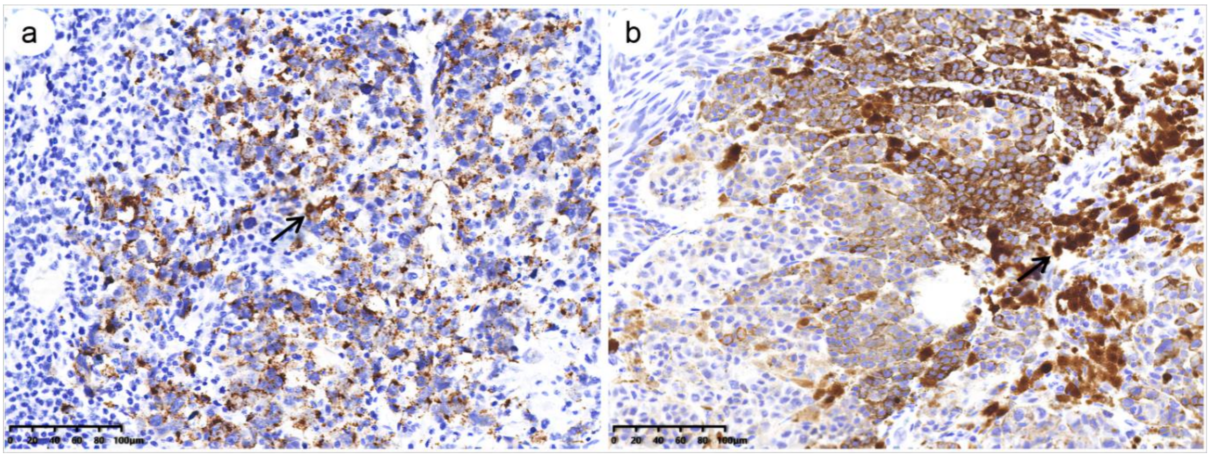

Fig S3: Immunohistochemical staining with Melan-A antibody (x2). The specimen displayed on the left side of the image represents a surgically excised esophageal mass, whereas the tissue on the right corresponds to melanoma tumor tissue.

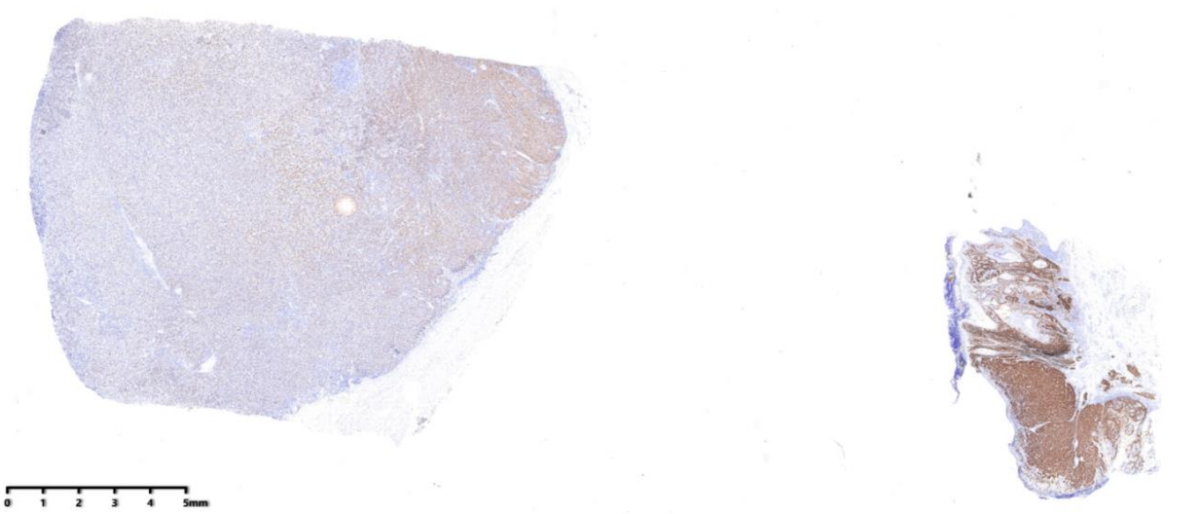

Fig S4: Immunohistochemical staining with Melan-A antibody (x40). (a) The esophageal mass (arrow). (b) The positive control of melanoma tumor tissue (arrow).

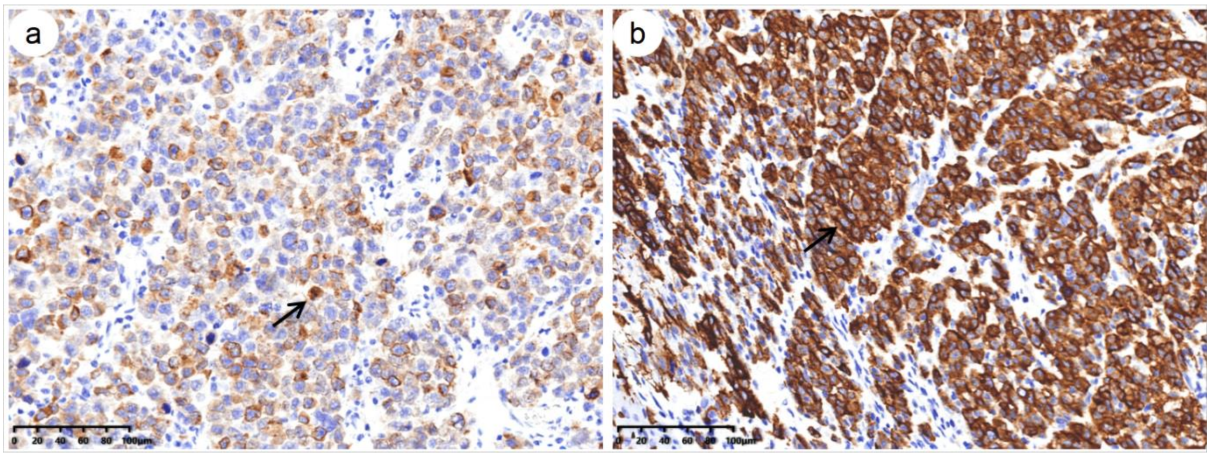

Supplement: Supplementary file 1 [file medi-104-e44988-s001.pdf]
